# A Novel Insight Into the Fate of Cardiomyocytes in Ischemia-Reperfusion Injury: From Iron Metabolism to Ferroptosis

**DOI:** 10.3389/fcell.2021.799499

**Published:** 2021-12-02

**Authors:** Jing-yan Li, Shuang-qing Liu, Ren-qi Yao, Ying-ping Tian, Yong-ming Yao

**Affiliations:** ^1^ Department of Emergency, The Second Hospital of Hebei Medical University, Shijiazhuang, China; ^2^ Translational Medicine Research Center, Medical Innovation Research Division and Fourth Medical Center of the Chinese PLA General Hospital, Beijing, China

**Keywords:** ischemia-reperfusion injury, cardiomyocyte, cell death, ferroptosis, iron metabolism

## Abstract

Ischemia-reperfusion injury (IRI), critically involved in the pathology of reperfusion therapy for myocardial infarction, is closely related to oxidative stress the inflammatory response, and disturbances in energy metabolism. Emerging evidence shows that metabolic imbalances of iron participate in the pathophysiological process of cardiomyocyte IRI [also termed as myocardial ischemia-reperfusion injury (MIRI)]. Iron is an essential mineral required for vital physiological functions, including cellular respiration, lipid and oxygen metabolism, and protein synthesis. Nevertheless, cardiomyocyte homeostasis and viability are inclined to be jeopardized by iron-induced toxicity under pathological conditions, which is defined as ferroptosis. Upon the occurrence of IRI, excessive iron is transported into cells that drive cardiomyocytes more vulnerable to ferroptosis by the accumulation of reactive oxygen species (ROS) through Fenton reaction and Haber–Weiss reaction. The increased ROS production in ferroptosis correspondingly leads cardiomyocytes to become more sensitive to oxidative stress under the exposure of excess iron. Therefore, ferroptosis might play an important role in the pathogenic progression of MIRI, and precisely targeting ferroptosis mechanisms may be a promising therapeutic option to revert myocardial remodeling. Notably, targeting inhibitors are expected to prevent MIRI deterioration by suppressing cardiomyocyte ferroptosis. Here, we review the pathophysiological alterations from iron homeostasis to ferroptosis together with potential pathways regarding ferroptosis secondary to cardiovascular IRI. We also provide a comprehensive analysis of ferroptosis inhibitors and initiators, as well as regulatory genes involved in the setting of MIRI.

## Introduction

To date, revascularization is commonly regarded as one of the efficacious treatments for ischemic cardiomyopathy in patients with critically acute myocardial infarction (Yang et al., 2018). However, reperfusion therapy is inevitably complicated with myocardial ischemia-reperfusion injury (MIRI), which is responsible for increased mortality and poor outcomes in myocardial infarction, thereby reducing the preponderance of reperfusion therapy to a large extent (Bell and Yellon, 2011). It has been demonstrated that ischemia-reperfusion injury (IRI) is especially involved in persistent impairment of cardiac function, followed by myocardial remodeling. Therefore, timely recognition and prompt interference for MIRI are the currently clinical incidents that need to be urgently resolved to improve the survival and prognosis of myocardial infarction patients. Dysfunction in cardiomodulatory response compromised by IRI impairs cardiometabolisms, including oxidative stress, systemic inflammation, calcium metabolic disorders, mitochondrial damage, and iron overload, which ultimately results in a vicious cycle between progressive disturbance of cardiomyocyte metabolism and irreversible myocardial remodeling (Turer and Hill, 2010). Deep insights into the interplay between IRI and intracellular metabolism as well as cell death in cardiomyocytes are of great importance in extending the knowledge of the pathogenesis and development of MIRI.

As we know, iron is considered an essential mineral that serves as a prerequisite in pivotal biological processes, including oxygen transfer, enzymatic catalyzed reaction, aerobic respiration, lipid peroxidation, and intracellular metabolism (Hirst, 2013). Iron deficiency jeopardizes the contractility of cardiomyocytes by subduing mitochondrial function and decreasing energy generation, resulting in impairment of cardiac function (Hoes et al., 2018). Upon the physiological state, iron is capable of playing an important role in energy metabolism through multiple ways to enter cardiomyocytes, being utilized for storage or transported into the mitochondrion to take part in biosynthetic reaction (Valko et al., 2016). Under the challenge of continuous stress or decompensatory response however iron reversely drives a poisonous property resulting from the overproduction of reactive oxygen species (ROS), which collapses the balance between generation and depletion in free radicals *via* the Fenton reaction and Haber–Weiss reaction (Valko et al., 2016). Uncontrolled accumulation of iron concentration and redox efficacy of ferrous (Fe^2+^) ions facilitate a perniciously metabolic network in cardiomyocytes, contributing to lipid peroxidation along with ROS overproduction, which exert a great threat to the function of basic cellular mechanisms (Gammella et al., 2016; He et al., 2019). Increased mitochondrial iron-related ROS generation is another regulatory contributor to both malfunction of intrinsic mitochondria and cardiac tissue injury. Mitochondrial iron is deemed as the major factor determining cardiomyocyte fate as evidenced by approximately one-third of cardiomyocyte iron reserves in the mitochondria. Moreover, it has been reported that the iron content in cardiomyocyte mitochondria is 50% higher than that of the other cells (Wofford et al., 2017). Increasing clinical studies have suggested that the level of cardiomyocyte iron is a prognostic factor of MIRI accounting for the deposition of iron in cardiac tissue in the occurrence of MIRI. Additionally, the impeded erythrocyte flow in the obstructive region might lead red blood cells to be lysed, resulting in the accumulation of iron from hemoglobin, which can eventually generate excessive ROS and trigger pathological events of MIRI. As is hypothesized that iron deposition might be recognized as an integral element of pathophysiology for triggering MIRI and increasing mortality risk, it is of great significance to clarify the underlying mechanisms of iron-mediated cell death to further provide potential targets for MIRI therapy.

As we all know, no matter what mechanisms are involved in the pathophysiology of MIRI, the final foreordination of the injured cardiomyocyte is the distinct forms of programmed cell death, which have been identified to play an essential role in MIRI, namely, apoptosis, necroptosis, pyroptosis, and ferroptosis. Over the past decades, regarded as the main types of cell death for cardiomyocytes, apoptosis is considered a form of programmed cell death, while necrosis is thought to be an accidental and uncontrolled type of cell death. In recent decades however, necrosis is precisely regulated by differently signaling pathways, including necroptosis, ferroptosis, pyroptosis, oxytosis, and parthanatos. Currently, noxious iron overload has been reported to induce non-apoptosis cell death, defined as ferroptosis, which presents with a deteriorative cross talk between lipid peroxidation, and a mass of ROS accumulation depended on excessive iron, together with the dominating causes of reduction in glutathione synthesis and deactivation of enzyme glutathione peroxidase 4 (GPX4) (Dixon et al., 2012). Indeed, multiple factors are involved in the pathogenesis of cardiac I/R injury implicated by ferroptosis, such as amino acid and lipid metabolism as well as iron mobilization and peroxidation through Fenton reaction (Fang et al., 2019). The regulatory effects of other factors, including activity of GPX4, ferroptosis-associated endoplasmic reticulum stress (ERS), and the mammalian target of rapamycin (mTOR)-mediated iron transport proteins, are also noteworthy but remain controversial (Baba et al., 2018; Li et al., 2020). From a molecular perspective, both ferroptosis-related initiators and inhibitors might be the potential targets for treatment of MIRI since ferroptosis accompanied by excess iron is confirmed to be the leading cause of cardiomyocyte death. Suppressing ferroptosis to prevent cardiac cell death and alleviate cardiac remodeling might become an efficacious therapeutic strategy for MIRI.

## Iron Homeostasis and Its Role in Cardiomyocytes

**FIGURE 1:**
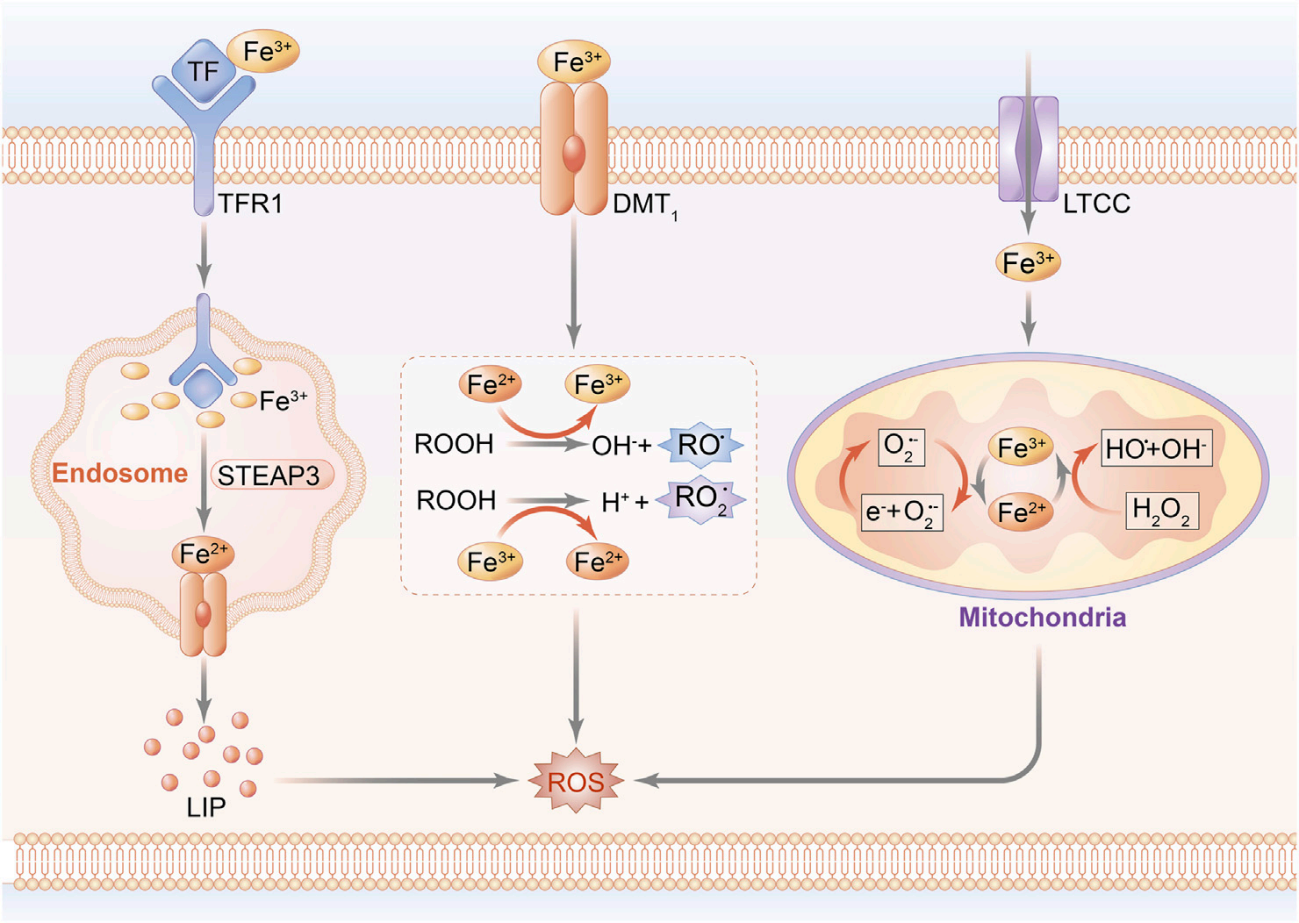
Schematic diagram of iron homeostasis in cardiomyocytes. Peripheral iron is bound with Tf and imported into the intracellular area through the transport of TfR1, while non-Tf–combined iron is transported by DMT-1 and LTCC. The uptake of iron is internalized by endocytosis. A part of iron mediates lipid peroxidation by catalyzing ROOH into the formation of RO and RO_2_ radicals. Other parts of Fe(II) and Fe(III) are transported into mitochondria to conduct peroxidative reaction through Fenton reaction, and eventually upregulate ROS generation.; Abbreviations: Tf, transferrin; TfR1: transferrin receptor 1; DMT-1, divalent metal transporter 1; LTCC, L-type voltage-dependent Ca^2+^ channels; ROOH, hydroperoxysides; RO, alkoxyl; RO_2_, peroxyl; and STEAP3, STEAP family member 3.

The pathophysiological progression of cardiomyocyte injury reportedly results from a disorder of iron homeostasis attributed to the overproduction of ROS and formation of the mitochondrial permeability transition pore (mPTP), ultimately leading to the induction of cardiomyocyte death (Morciano et al., 2015; Pell et al., 2016). In fact, hyperactivation of hypoxia-inducible factor (HIF) upregulates the mitochondrial ferritin (FtMt) expression of transferrin receptor 1 (TfR1), followed by increased iron accumulation, which traps cardiomyocytes into a vicious cycle of exacerbated ROS-induced impairment (Tang et al., 2008). A unique cohort of FtMt, featured on ferroxidase activity and regulatory capacity of iron metabolism in mitochondria, acts as a prime contributor to myocardial vitality and ROS regulation in IRI (Wood, 2008; Wu et al., 2016). Since most of mitochondrial ROS arise from redox reaction in the initial stage of oxidative stress, it is reasonable to predicate that mitochondrial function serves as a bridge connecting iron homeostasis to ROS production during the occurrence of MIRI, suggesting that reversal of mitochondrial iron–associated peroxidation reaction might be one of the beneficial interferences with MIRI. Notably, the schematic diagram of iron homeostasis in cardiomyocytes and mitochondria is shown in Figure 1.

### Iron Transport in Cardiomyocytes

Cardiomyocyte intussuscepts iron mainly depending on ferritin (FT), either by means of combination with TfR1 and subsequently receptor-mediated endocytosis, or through the calcium channels and zinc transporters in the cardiac plasma membrane, which are mediated by divalent metal transporter 1 (DMT-1) protein (Hentze et al., 2010). In addition to being stored in FT cores in normal circumstances, ferrous ion can be mobilized and released to peripheral circulation *via* ferroportin (FPN), which is located on the basolateral membrane of enterocytes (Oudit et al., 2003). Upon entrance into the cardiomyocytes, iron is stored in the labile iron pool (LIP), where the level of iron is maintained stably on a normal range but abruptly increases under exposure to the pathological state, acting as an intermediator to promote heme and iron–sulfur cluster production in the mitochondrion through the biosynthetic pathway (Lane et al., 2015). Iron in LIP can be utilized for storage in FT, which serves as a ubiquitous intracellular buffer to prevent iron deficiency and iron overload, contributing to its efficacy in reserving as much as 5,000 atoms of iron in a soluble form and transporting iron to the requisite site (Watt, 2013). FT, composed of 24 subunits of both ferritin heavy chain (FTH) and ferritin light chain (FTL), is regarded as a key factor implicated with iron homeostasis for the reason that it combines and segregates iron in the case of redundant iron importing to protect against oxidative stress, whereas it releases and transports iron when suffering iron deficiency (Kawabata, 2019).

Notably, intracellular iron concentration and homeostasis are affected by several key regulators. Since the peroxidative peculiarity of iron prompts it to be a redox catalyst for the generation of noxious ROS, intracellular iron is exactly regulated by the ROS-dependent cell signaling pathway to maintain biological function. One of these catalytic responses is initiated in the Fenton reaction, for example, stimulating hydroxyl radical (HO) production, and another contributor concentrates on ferrous ion–mediating lipid peroxidation response, in turn inducing the accumulation of lipid radicals (Aisen et al., 2001). Moreover, transcriptional modification after the interconnection between iron regulatory proteins (IRPs) and iron responsive elements (IREs) is involved in the regulated mechanisms with regard to the cellular iron homeostatic process in iron intake, reservation, and release by modulating the synthetic function of iron metabolism–associated proteins (Haddad et al., 2017). Under the low content of cellular iron, bidirectional IRPs are responsible for either stabilizing the mRNA expression of TfR1 and DMT-1 to facilitate iron indrawal, or conducting a suppressed impact on mRNA translation of FT in order to inhibit iron storage (Paterek et al., 2019). Additionally, the degradation of FPN has been proven to be independently related to hepcidin in cardiomyocytes, and it accounts for the inactivation of FPN, eventually resulting in reduced iron release. Actually, cardiac hepcidin is found to be a major cause for iron metabolism in cardiomyocytes, and it exhibits a distinction from systemic iron regulation due to its autologous secretive function that promotes cardiac hepcidin protein upregulation rather than downregulation onset of hypoxia (Lakhal-Littleton et al., 2016). Thus, the fact that only one FPN protein is available for iron exporting during iron accumulation might further confirm cardiomyocytes to be more sensitive to iron overload than other cell types.

### The Role of Iron Metabolism in Myocardial Mitochondria

The mitochondrion, known as an essential organelle for systemic energy metabolism, confers an important impact on iron homeostasis and modulation of myocardial damage during IRI (Richardson et al., 2010; Lesnefsky et al., 2017; Vela, 2020). It has been documented that mitochondria provide available sites for heme synthesis and iron–sulfur cluster (ISC) generation, with formation of heme and ISC proteins to be integrated in the mitochondrial oxidative phosphorylation system, which supplies a physiological necessity of cardiac activity for continuous energy through catalyzing electron transport of oxidative iron (Paul et al., 2017). Thus, iron concentration in mitochondria is closely related to the fate of cardiomyocytes since the level of mitochondrial iron in cardiac myocytes is remarkably higher than other cells. As reported by Wofford et al., insufficient iron might impose restriction on energy export, whereas uncontrolled iron overload could lead to a disruption of the mitochondrion *via* redundant ROS generation (Kruszewski, 2003; Wofford et al., 2017). Along with ROS-associated toxicity in mitochondria, the productions of poisonous hydroxyl radicals, as a result of the reaction between ROS and mitochondria, contribute to depolarization in mitochondrial membrane potential (MMP) and openness in the mitochondrial penetrability pore, thereby leading to aberrant morphology of mitochondrial swelling as well as mitochondrial dysfunction (Sripetchwandee et al., 2014; Chan et al., 2018).

Despite explicit mechanisms concerning iron transportation through mitochondrial ectoblast remain to be ascertained, one of the most evident studies suggests that both the Tf-TfR complex and FT degradation in the lysosome are the major sources of importing iron from the cytoplasm to the mitochondrion, which is regulated by mitoferrin and mitochondrial calcium uniporter (Gordan et al., 2018). FtMt, another key regulator of mitochondrial iron homeostasis, especially expressing on cardiomyocytes and possessing a highly homologous sequence with FTH, conducts a pleiotropic effect on iron input by means of redistribution of iron from the cytosol to the mitochondrion (Santambrogio et al., 2007). On account of this regulatory mechanism, elevated expression of FtMt on cardiomyocytes is found to be a major cause for reduction of iron in mitochondrial LIP, subsequently resulting in decreased systemic ROS generation (Nie et al., 2005; Campanella et al., 2009). Further evidence has shown that the overexpression of FtMt significantly inhibits erastin-induced ferroptosis due to its impact on decreased ROS production (Wang et al., 2016). Of note, FtMt might be a potential target for maintaining iron homeostasis in cardiomyocytes.

## Molecular Mechanisms of Iron Metabolism in Cardiomyocyte IRI

Iron metabolism imbalance, especially iron overload, has been demonstrated to be implicated in the pathology of cardiomyocyte IRI, resulting from the driving forces of excessive ROS and oxygen free radicals that attribute to antioxidant system disrupt onset of constant exposure to IRI (Zhou et al., 2015; Cadenas, 2018). Inherently, this pathological process further aggravates oxidative stress accompanied by myocardial membrane damage and cardiovascular endothelial dysfunction (Dongó et al., 2011). In the early stage of ischemia and reperfusion, intracellular iron is conductive to be released in the acid internal environment of cardiomyocytes, augmenting iron-mediated Fenton reaction, which converses hyporeactive hydrogen peroxide to hyperreactive hydroxyl radicals (Williams et al., 1991). In this case, the administration of iron inhibitors at the initial phase of reperfusion might diminish free radical generation and attenuate cardiomyocyte IRI (Drossos et al., 1995).

### The Pathophysiology of Cardiomyocyte IRI

**FIGURE 2:**
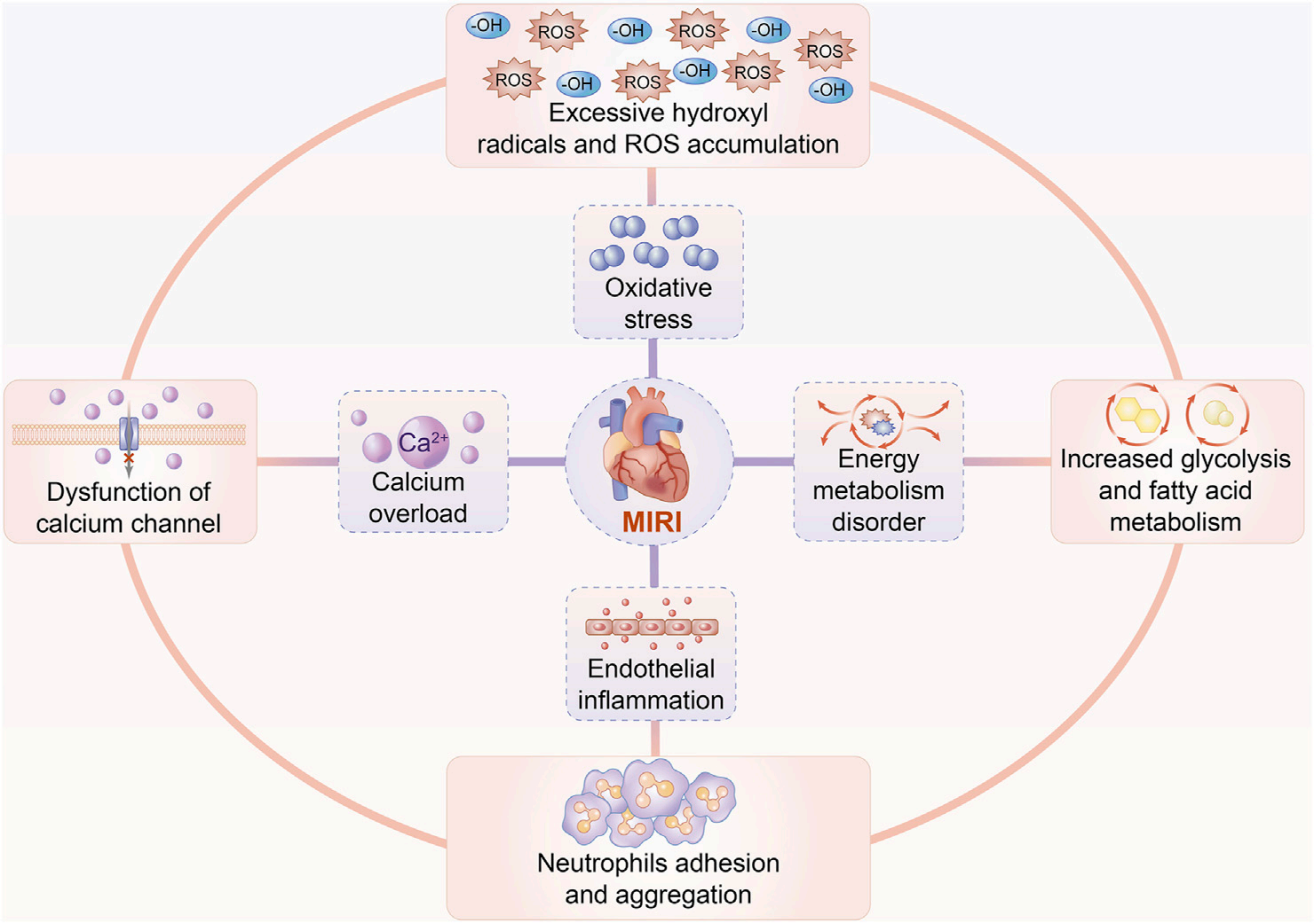
Pathophysiology of cardiomyocyte IRI. Multiple factors are involved in the pathogenesis and progression of MIRI, including oxidative stress, endothelial cell inflammation, calcium overload, and energy metabolic disturbance. Excessive ROS and hydroxyl radicals are produced to trigger peroxidative stress. Chemotaxis, adhesion, and aggregation of neutrophils initiate inflammatory cytokine release, in turn inducing endothelial cell injury. Calcium overload suppresses excitability and contractility of cardiomyocytes due to the dysfunction of the L-type calcium channel as well as the ryanodine receptor. Increased glycolysis and fatty acid metabolism, as well as mitochondrial dysfunction, ultimately deteriorate the disorder of energy metabolism.

Increasing evidence indicates that multiple pathophysiological factors are involved in the development of MIRI, including oxidative stress, endothelial cell inflammation, calcium overload, and energy metabolism disorder. Cardiomyocyte IRI is commonly accompanied by redundant oxygen-free radicals and accumulated ROS after cardiovascular reperfusion, which finally evokes the peroxidation of proteins, lipids, and nucleic acids. These endogenous superoxide products can further accelerate membrane injury and organelle dysfunction (Matsushima et al., 2014). Along with hyperactivated inflammation compromised by conglutination and infiltration of neutrophils, cardiomyocyte IRI unexpectedly worsens as a result of robust feedback on increased metabolism of arachidonic acid, in turn leading to a massive production of inflammatory cytokines (Vinten-Johansen et al., 2007; Boag et al., 2017). During cardiomyocyte I/R injury, high levels of pro-inflammatory cytokines promote either the myocardial tissue to maintain a pro-inflammatory state or endothelial cells to induce autophagy, eventually exposing cardiomyocytes to a more vulnerable damage in structure and function (Russo et al., 2017; Schanze et al., 2019). Intracellular calcium, with the peculiar capacity of maintaining cardiomyocyte functions, appears to be more sensitive to reperfusion, and it is largely overloaded when cardiomyocytes suffer IRI (Verkhratsky and Parpura, 2014). Similar to excessive amounts of iron, calcium overload suppresses excitability and contractility of cardiomyocytes, attributed to the combination between calcium and troponin, which finally debilitates the contraction of myocardial cells (Grueter et al., 2006). With regard to energy metabolism during cardiomyocyte IRI, ATP produced by glycolysis is considered to be the primary source of energy for the maintenance of cardiomyocyte vitality at the initial phase of reperfusion. Inevitably, this action causes cardiomyocytes to suffer a vicious cross talk between lactate accumulation and the acidic environment, resulting in fatty acid peroxidation together with an energy metabolism disorder (Tian et al., 2019). Taken together, cardiomyocyte IRI depends on the energy metabolism disorder initiated in the ischemia-hypoxia setting and enhances production of oxygen free radicals, which indirectly induce calcium along with inflammation, leading to mitochondrial damage **(** Figure 2 **)**.

### The Mediators of Iron Metabolism in Cardiomyocyte IRI

It has been reported that multiple iron metabolism–associated factors are thought to be associated with the pathogenesis of cardiomyocyte IRI, including HIF and FTH signaling pathways and mitochondrial iron protein–regulated pathway (Table 1). The HIF, for instance, presents with visible hyperactivation, which, in turn, upregulates iron TfR1 expression and causes iron overload during cardiomyocyte IRI, eventually exasperating ROS-induced peroxidative injury (Zhang et al., 2019a). Further evidence has shown that the administration of cardiomyocytes with iron chelators after I/R injury is beneficial for the reversion on myocardiac malfunction (Paraskevaidis et al., 2005). Another mediator of FTH, as observed trending a down-expressed toward in the mouse model of MIRI, exerted a suppressed effect on the capacity of cardiomyocytes in binding free iron, leading to oxidative stress and even cell death (Omiya et al., 2009). Unique cohorts of mitochondrial iron proteins are demonstrated to complicate modulating cardiomyocyte IRI and play important roles in survival and prognosis of cardiomyocytes *via* controlling ROS generation. For instance, mitochondrial iron import is regulated by mitoferrin 2 (MFRN) and mitochondrial calcium uniporter (MCU), whereas its export is modulated *via* ABCB proteins (Ichikawa et al., 2012). The same verification goes for ATP-binding cassette subfamily B member 8 (ABCB8) that upregulated expression of ABCB8 through genetic modification is identified to effectively promote mitochondrial iron output and finally protect cardiomyocytes against IRI (Chang et al., 2016). Hence, mitochondrial iron regulators might become effective targets for improving outcomes in the setting of MIRI (Chang et al., 2016).

**TABLE 1:** Mediators of iron metabolism in MIRI-associated diseases.

Molecule	Regulatory effects
HIF	Enhances TfR1 expression and exasperates iron overload and ROS production
FTH	Binds iron and suppresses cardiomyocyte capacity
MFRN	Regulates mitochondrial iron import
MCU	Regulates mitochondrial calcium uniporter
ABCB8	Augments mitochondrial iron output
IRP	Modulates iron intake, reservation, and release
IRE	Regulates the synthesis of iron metabolism–associated proteins
FPN	Mobilizes and releases ferrous ion to peripheral circulation
FtMt	Inputs iron and redistributes iron from the cytosol to mitochondria
DMT-1	Promotes iron indrawal and inputs iron

Abbreviations: HIF, hypoxia-inducible factor; FTH, ferritin heavy chain; MFRN, mitoferrin 2; MCU, mitochondrial calcium uniporter; ABCB8, ATP-binding cassette subfamily B member 8; IRP, iron regulatory proteins; IRE, iron-responsive elements; FPN, ferroportin; FtMt, mitochondrial ferritin; and DMT-1, divalent metal transporter 1.

## Ferroptosis in Cardiomyocytes After IRI

**FIGURE 3:**
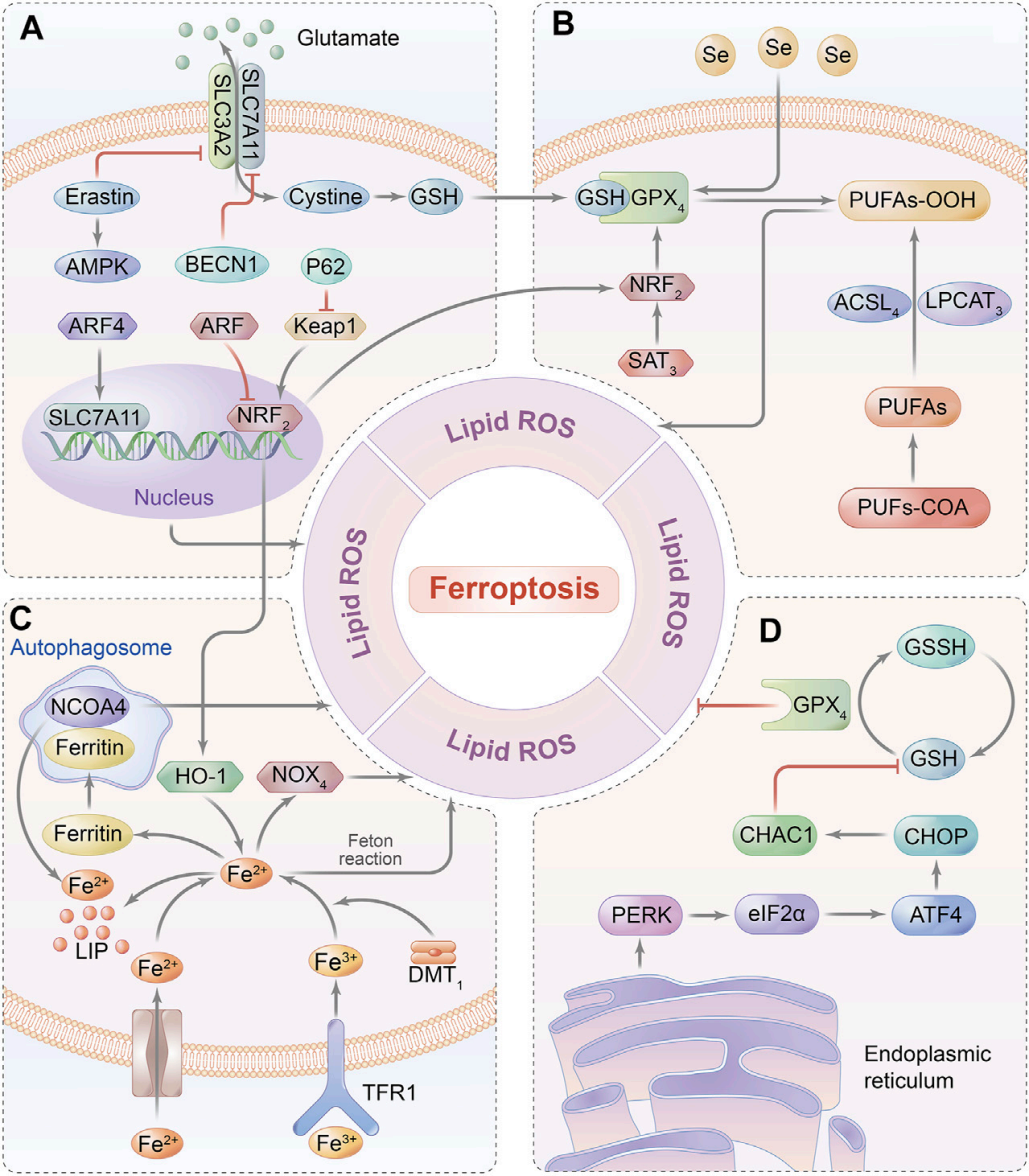
Ferroptosis-related signaling pathway. **(A)** GSH relies on the molecular substrate called cysteine, which is transferred *via* a heterodimeric cell membrane antiporter, system Xc-that is composed of SLC7A11 and SLC3A2, regulating ferroptosis by exchanging glutamate and cystine at a 1:1 ratio. **(B)** Ferroptosis is triggered by the peroxidation of PUFAs and accumulation of ROS, which are catalyzed by ACSL4 and LPCAT3. GPX4 can hydrolyze lipid peroxides into innocuous alcohols. **(C)** Free iron bound with Tf is transported into intracellular area *via* TfR1 in endosomes. Fe (III) is reduced to redox-active iron (FeII) by ferrireductase of STEAP3, while Fe (II) is released from endosomes into LIP through DMT1. Under the oxidative stress, Fe^2+^ catalyzes the generation of hydroxyl radicals by Fenton reaction, which eventually initiates ferroptosis. Ferritin is utilized to iron storage and can be degraded by NCOA4-mediated ferritinophagy. **(D)** GSH synthesis is a requisite for GPX4 in mediating an antioxidant impact, thereby inhibiting the iron-dependent ROS production by catalyzing lipid hydroperoxides into lipid alcohols.; Abbreviations: GPX4, glutathione peroxidase 4; PUFAs, poly-unsaturated fatty acids; NCOA4, nuclear receptor coactivator 4; GSH, glutathione; SLC7A11: solute carrier family 7 member 11; SLC3A2, solute carrier family 3 member 2; ACSL4, acyl-coA synthetase long-chain family member 4; LPCAT3. lysophosphatidylcholine acyl-transferase 3; Tf, transferrin; TfR1, transferrin receptor 1; ATF4, activating transcription factor 4; and CHOP, C/EBP homologous protein.

Elevated concentration of the intracellular iron–induced poisonous ROS-dependent reaction is defined as ferroptosis, which is a non-apoptotic programmed cell death and is characterized by overload iron-associated lipid peroxidation (Conrad et al., 2018). It can be initiated by either depletion of glutathione biosynthesis or inactivation of the antioxidant enzyme GPX4 that is deemed a consequence of iron-relevant ROS generation as well as polyunsaturated fatty acid (PUFA) peroxidation, contributing to the disruption of redox homeostasis (Dixon et al., 2012; Cao and Dixon, 2016). Currently, the major signaling pathways of ferroptosis are summarized in Figure 3. Moreover, recent studies have confirmed ferroptosis to be a substantial contributor to the pathogenesis of cardiomyocyte IRI, as evidenced by the protective effect on cardiomyocytes once the ferroptosis inhibitor is administered (e.g., liproxstatin-1, Lip-1) on a MIRI model, implicating that ferroptosis might provide a novel treatment targeted for diseases associated with cardiomyocyte IRI (Díez-López et al., 2018). Therefore, the effects of ferroptosis-related molecular pathways on MIRI are as follows.

### Iron Metabolism Signaling Pathway

Iron overload resulted from either cytoplasm iron imbalance or mitochondrial iron disturbance exerts an impact on pathogenesis and progression of ferroptosis. One of the intracellular iron modulators nuclear receptor coactivator 4 (NCOA4), for example, can be regulated by ferritinophagy to further suppress transferrin exporting intracellular iron, while another key encoder named iron-responsive element-binding protein 2 (IREB2) mainly affects transferrin expression as well as iron transportation (Xie et al., 2016). Likewise, the cross talk of iron on the mitochondrial membrane is primarily regulated by the voltage-dependent anion channel (VDAC) located on the outer mitochondrial membrane (Tateda et al., 2012). VDAC 2/3 shows evident signs of opening under persistent exposure to mitochondrial iron accumulation, and this subsequently primes the response of ferroptosis *via* regulating the upstream protein of FtMt. Accordingly, the overexpression of FtMt can intercept mitochondrial iron to protect cells from ferroptosis (Maldonado et al., 2013).

### Glutathione Metabolism Signaling Pathway

Intracellular reduced glutathione (GSH), considered the main antioxidant buffer, commonly displays the ability in protecting against lipid peroxidation in ferroptosis by donating an electron to GPX4, which can suppress the formation of iron-dependent ROS by transforming lipid hydroperoxides into lipid alcohols (Stockwell et al., 2017; Ingold et al., 2018). Originally, endogenous biosynthesis of GSH relies on the molecular substrate called cysteine, which is transferred *via* a heterodimeric cell membrane antiporter, system Xc- that is composed of a transmembrane protein transporter solute carrier family 7 member 11 (SLC7A11) and a single-pass transmembrane regulatory protein solute carrier family 3 member 2 (SLC3A2), regulating ferroptosis by exchanging glutamate and cystine at a 1:1 ratio (Fujii et al., 2019). Importantly, the ferroptotic inhibitor of erastin can directly restrain system Xc- function resulting in GPX4 hypoactivation due to the depletion of GSH (Hayano et al., 2016). Consistent with this finding, the knockdown of GPX4 could exasperate lipid peroxidation–mediated ferroptosis, suggesting GPX4 to be the key negative regulator of ferroptosis (Gladyshev et al., 1999).

### Lipid Peroxidation Signaling Pathway

Even though the systemic antioxidant effect dominates the enzyme-linked reactions, long-term exposure to oxidative stress can inevitably trigger the biosynthesis of PUFAs in decompensation by activating lipid metabolism–related enzymes, including acyl-coA synthetase long-chain family member 4 (ACSL4) and lysophosphatidylcholine acyl-transferase 3 (LPCAT3) (Anthonymuthu et al., 2018). PUFAs are catalyzed to generate subversive lipid peroxides that destruct cell morphology, such as mitochondrial membrane shrinkage and membrane imperfection, ultimately leading to ferroptosis (Ng et al., 2020). In accordance with these findings, exogenous unsaturated fatty acids might not only inhibit the susceptibility of cells to ferroptosis but also refrain the lipid bilayer of the cell membrane from peroxidative injury (Magtanong et al., 2019).

### NOX4 Signaling Pathway

Previous studies have regarded NADPH oxidase 4 (NOX4) to be the major molecular medium of oxidative stress in cardiomyocytes that transfers electrons from NADPH to the oxygen atom, even producing superoxide (Kuroda et al., 2010). On account of its inductive ability in peroxidation, NOX4 deficiency inhibits the cardiomyocytotoxicity and mitochondrial injury induced by produced intracellular free radicals, implying that NOX4 is compromised in oxidative stress-mediated cell damage (Sun et al., 2017). As discovered by Chen et al., the knockdown of NOX4 dramatically reversed ventricular remodeling by means of improving iron overload in the heart failure model, which further inferred pharmaceutical inhibition or knockout of GPX4 to be effective in precaution of ferroptosis (Chen et al., 2019). Remarkably, apoptosis-inducing factor mitochondrion-associated 2 (AIFM2, also named FSP1) is another electronic carrier and lipid-soluble antioxidant independent of GPX4, which acts as a nicotinamide adenine dinucleotide phosphate (NADP)–dependent coenzyme Q (CoQ) oxidoreductase to suppress ferroptosis by directly modulating the CoQ antioxidant system (Marshall et al., 2005).

### ATF4 Signaling Pathway

Activating transcription factor 4 (ATF4) has been documented to involve in the regulation of autophagy, oxidative stress, and inflammatory response, and partially expresses at a low level under the normal condition, whereas it is dramatically overexpresses upon stimulation with hypoxia or ERS (Pitale et al., 2017). A recent report revealed that activation of the ATF4-C/EBP homologous protein (CHOP) signaling pathway was closely related to ferroptosis-associated diseases such as cardiomyocyte IRI by modulating the ATF4-targeting gene of CHAC1 to induce GSH degradation (Chen et al., 2017a; Wang et al., 2019; Li et al., 2020). Based on the capacity of heat shock protein 5 (HSPA5) in mediating endoplasmic reticulum unfolding of the protein to negatively regulate GPX4, induction of HSPA5 expression can feedback on the upregulation of ATF4 activity to elevate the GPX4 level, eventually preventing cells from ferroptosis (Bai et al., 2020). Therefore, cascaded signals of ATF4-HSPA5-GPX4 implicated in oxidative response and the metabolic system might act as a negative feedback on ferroptosis *via* complex networks (Zhu et al., 2017).

### NRF2 Signaling Pathway

A transcription factor in terms of nuclear factor erythroid 2–related factor 2 (NRF2) can mechanistically regulate ferroptosis by not only promoting iron storage to reduce iron accumulation, but also upregulating SLC7A11 activity to increase glutamate content (Fan et al., 2017; Kerins and Ooi, 2018; Mou et al., 2019). Meanwhile, NRF2 is modulated by upstream molecules, such as P62 and alternative reading frame (ARF). P62 likely suppresses the degradation of NRF2 to further enhance nuclear reservation, whereas ARF directly inhibits transcriptional function targeting to downregulate SLC7A11 expression; both of them effectively prevent ferroptosis (Sun et al., 2016; Chen et al., 2017b). Likely, ferroptosis-associated FT, together with heme oxygenase (HO-1), is affected by NRF2 as evidence showed that HO-1 knockdown deteriorated erastin-induced ferroptosis, but NRF activation might inversely upregulate HO-1 (Gai et al., 2020). Collectively, NRF2 is identified to be the main negative mediator in signaling cross-connection for protecting the cell against ferroptosis.

**FIGURE 4:**
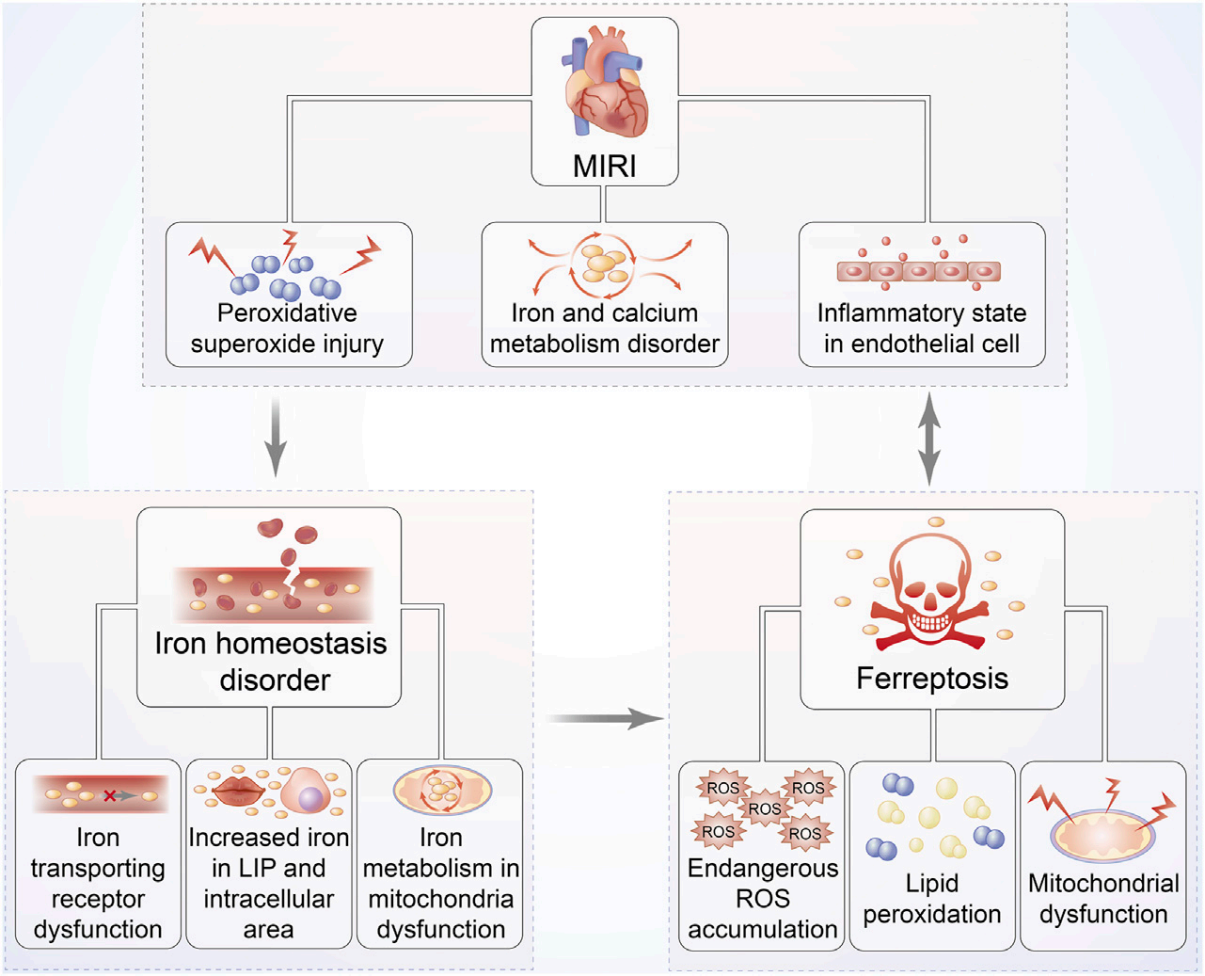
Relationship between MIRI and iron homeostasis disorder as well as ferroptosis. The pathophysiological progression of MIRI is closely associated with iron homeostasis disorder, overproduction of ROS, and mitochondrial dysfunction, followed by increased iron accumulation. These responses can further trap cardiac cells into a vicious cycle of exacerbated ROS-induced impairment, eventually leading to ferroptosis. Hearts suffering I/R injury are exposed to persistent oxidative stress, especially iron-dependent lipid hydroperoxide generation, which might damage the contractility of cardiomyocytes.

Since the viewpoint that abundant iron deposited on cardiac cells in the MIRI mouse model to induce ferroptosis was first recognized by Bata et al., it led us to further investigate the link between ferroptosis and cardiomyocyte IRI (Baba et al., 2018) **(** Figure 4 **)**. Oxidative stress plays the predominant role in cardiomyocyte IRI that is manifested by chronic inflammation in vascular walls and lipid peroxidation deposition in the arterial wall (Berliner, 2002). Theoretically, membrane phospholipid compounds of PUFAs exhibit high susceptibility to esterification as evidenced by the signs on phospholipid oxidation products and subsequent damage *via* the production of ROS onset of MIRI (White et al., 2015). With continually increasing species of oxidized lipoacylcholine being discovered during cardiomyocyte IRI, the mechanisms underlying lipid peroxidation have been confirmed to be the connection between ferroptosis and MIRI (Yeang et al., 2019). Moreover, the exogenous supplement of these oxidized lipid products to cardiomyocytes can definitely trigger ferroptosis-associated cell death; thus, ferroptosis regulates cardiomyocyte IRI by affecting phospholipid metabolism (Ganguly et al., 2018).

The iron-related signaling pathway is recognized to be another prerequisite for ferroptosis-mediated cardiomyocyte IRI secondary to lipid oxidative stress, which might be attested by the protective effect of the iron chelator called deferoxamine in suppressing cardiac cells from ferroptosis by means of binding iron in an MIRI model (Kakhlon and Cabantchik, 2002; Gao et al., 2015). A previous study confirmed iron to be an indispensable substrate for NADPH oxidase that was capable of catalyzing to produce superoxides, finally initiating ferroptosis (Dixon and Stockwell, 2014). Consistently, ferroptosis-mediated upregulation of NADPH oxidase and monocyte adhesion might account for cardiomyocyte IRI due to endothelial dysfunction following reperfused stress, hinting that the iron metabolism–associated pathway belongs to the leading cause for ferroptosis involved in MIRI (Sullivan, 2009; Kuo et al., 2014). The aforementioned notions were further validated by the subsequent fluorescence confocal analysis of cardiac muscle tissue from the MIRI mouse model, which manifested redundant iron accumulation and even non-apoptotic cell death of ferroptosis, showing an improved appearance after administration of ferrostatin-1 (Fer-1) (Stamenkovic et al., 2021). Moreover, rapamycin, discovered remarkably overexpressed in the early stage of MIRI, was identified to be the target for multiple iron transport proteins, which could modulate transferring receptor to upregulate ferroportin expression, suggesting that mTOR was implicated in the mechanistic process of ferroptosis-modulating MIRI (Bayeva et al., 2012; Guan and Wang, 2014). These documentations suggest that ferroptosis regulates MIRI *via* the iron metabolic signaling pathway, and the ferroptotic inhibitor of Fer-1 might be a potential approach to attenuate reperfusion damage.

Notably, some other ferroptotic-relevant molecules participate in the pathogenesis and progression of MIRI, including GPX4- and ERS-associated proteins. Lip-1, for instance, can prevent murine cardiomyocytes from IRI by inhibiting ferroptosis by enhancing GPX4 expression, and similarly another ferroptotic inhibitor of Fer-1 is found to alleviate MIRI of diabetes under constant hyperglycemia by weakening ERS (Li et al., 2019). These results suggest that GPX4, especially in GSH metabolism, as well as the ERS-mediated pathway may be driving factors for ferroptosis in regulating MIRI that need to be further clarified.

## Initiators and Inhibitors of Ferroptosis as the Potential Targets for MIRI

### Initiators of Ferroptosis

Depending on whether directly targeting for GPX4 activity, all the ferroptotic initiators are classified into two categories (Table 2). One type of inducers shows efficacy in inhibiting GPX4 activity by GSH depletion including erastin, sulfasalazine, diphenyleneiodonium chloride 2 (DPI2), buthionine sulfoximine (BSO), and lanperisone, while another form of inducers mainly directly blocks GPX4 without GSH consumption, such as RSL3 and DPI family except for DPI2. Mechanistically, erastin triggers ferroptosis either by combining mitochondrial VDAC2/3 to disrupt the respiratory chain together with ROS accumulation or weakening Xc system activity to attenuate GSH concentration accompanied by GPX4 inactivation (Yagoda et al., 2007; Yang et al., 2014). RSL3, impervious to the upstream of GPX4 including GSH depletion and cysteine intussuscepts, is able to inactivate GPX4 by the binding protein site, in turn augmenting ROS generation to initiate ferroptosis (Huang et al., 2021). On account of the succedaneous antioxidant approach upregulated by blocking GSH synthesis, BSO, owning perciclular capacity in suppressing peripheral GSH biosynthesis and eventually inactivating GPX4 to induce ferroptosis, exhibits lower efficacy on occasioning ferroptosis-related chain reaction than RSL3 (Harris et al., 2015). Although lanperisone can effectively enhance ROS production mediated by the RAS/MEK/ERK signaling pathway to induce ferroptosis due to KRAS gene mutation in embryonic fibroblasts, it displays less competency than erastin in resisting against KRAS-driven tumor vitality (Shaw et al., 2011).

**TABLE 2:** Ferroptosis initiators and inhibitors: targeting strategy for MIRI.

Reagent	Effects	Targets	Key mechanisms	References
Erastin	Induction	System Xc-	GSH depletion and GPX4 inactivation	Yagoda et al. (2007); Yang et al. (2014)
RSL3	Induction	GPX4	Binds GPX4 protein and increases ROS generation	Huang et al. (2021)
BSO	Induction	System Xc^-^	Suppresses peripheral GSH biosynthesis	Harris et al. (2015)
Lanperisone	Induction	System Xc^-^	Promotes ROS production	Shaw et al. (2011)
Sorafenib	Induction	System Xc^-^	Interdicts GSH biosynthesis	Zhang et al. (2013); Louandre et al. (2015)
Sulfasalazine	Induction	System Xc^-^	Downregulates SLC7A11 expression	Sehm et al. (2016)
DPI 7,10, 12	Induction	GPX4	Binds GPX4 and augments ROS production.	Sharma and Flora, (2021)
Ferrostatin-1	Inhibition	ROS	Prevents ROS generation	Yin et al. (2011)
Liproxstatin-1	Inhibition	ROS	Prevents lipid peroxidation	Zhang et al. (2019b)
Zileuton	Inhibition	5-Lipoxygenase	Inhibits PUFAs catalyzing into hyperoxide	Kagan et al. (2017)
Vitamin E	Inhibition	5-Lipoxygenase	Inhibits PUFAs catalyzing into hyperoxide	Liu et al. (2015)
Deferoxamine	Inhibition	Fe^2+^	Chelates intracellular superfluous iron	Doll and Conrad, (2017)
XJB-5-131	Inhibition	ROS	Eliminates poisonous ROS	Doll and Conrad, (2017)
Mitoquinone	Inhibition	ROS	Eliminates mitochondrial ROS	Fuchs and Steller, (2011)

Similar to the reagent of erastin, sorafenib, a multikinase inhibitor utilized for hepatic cancer therapy, interdicts GSH biosynthesis, rather than Raf suppression, and induces ferroptosis in tumor cells manifested as attenuation in tumor angiogenesis and restriction in tumor proliferation (Zhang et al., 2013; Louandre et al., 2015). Sulfasalazine authorized by the drug institution for nonbacterial inflammatory treatment was also confirmed to be applied to induce ferroptosis in gliofibroma cells by downregulating SLC7A11 expression (Sehm et al., 2016). Except for DPI2 which initiates ferroptosis conformed to erastin-associated mechanism, other members of DPI directly induce ferroptosis targeting for GPX4, instead of GSH reduction (Sharma and Flora, 2021).

### Inhibitors of Ferroptosis

Most varieties of ferroptotic inhibitors consist of antioxidants (e.g., Fer-1, Lip-1, and vitamin E), iron chelators (e.g., deferoxamine), and ROS scavengers (e.g., N-acetyl-l-cysteine, XJB-5-131, JP4-039, and mitoquinone), and they can potentially resist ferroptosis induced by RSL3 or erastin (Friedmann Angeli et al., 2014; Boonnoy et al., 2017; Zilka et al., 2017; Skouta et al., 2014; Krainz et al., 2016) (Table 2). Commonly, both Fer-1 and Lip-1, failing to suppress peroxidase activity, are able to capture free radicals to ameliorate ROS deposition, rather than inhibit ferroptosis as evidenced by the beneficial impact on the model of I/R injury (Yin et al., 2011; Zhang et al., 2019b). Based on the oxidant function of LOX in catalyzing unsaturated fatty acids into hyperoxides, the LOX inhibitor of zileuton and vitamin E antioxidant together with tocotrienol might protect cells from ferroptosis-induced oxidative stress (Liu et al., 2015; Kagan et al., 2017). Additionally, pretreatment of cells with deferoxamine, which chelates intracellular superfluous iron, may contribute to protecting cells against ferroptosis by disturbing the ROS-mediated Fenton reaction (Doll and Conrad, 2017). Currently, novel synthetic compounds defined as ROS scavengers, including XJB-5-131 and JP4-039, are reported to markedly suppress ferroptosis *via* eliminating poisonous ROS. In fact, the mitochondrion is considered the first line of defense in ROS clearance, and consequently, mitoquinone targets for mitochondrial ROS elimination (Fuchs and Steller, 2011).

## Conclusion and Perspectives

Cardiomyocyte IRI is commonly yet severely complicated by myocardial reperfusion intervention but is prone to be neglected in the clinical practice. The interplay between disorder of energy metabolism and massive accumulation of oxygen free radicals drives cardiomyocytes into a vicious circle in the setting of MIRI. Together with vascular endothelial inflammation, oxidative stress depending on iron homeostasis imbalance can damage cardiac function due to myocardial remodeling. Therefore, early interference of MIRI plays a critical role in the survival and prognosis of myocardial ischemia. Despite mechanisms with regard to ferroptosis in MIRI diseases being continually explored in experimental research studies and many progresses being achieved in clinical practice, certain limitations however remain to be overcome. First, precise downstream molecules of the signaling pathway in lipid peroxidation regulating ferroptotic myocardial remodeling appear unclear. Second, the potential mechanism of ferroptosis to activate the systematic inflammatory response in MIRI needs to be further explored. Third, more reasonable clinical trials are essential to be conducted in order to verify the outcomes in the established animal models of ferroptosis-related MIRI. In summary, ferroptosis appears to play an important role in pathogenic progression of MIRI; thus, targeting ferroptosis might provide a potential therapy for MIRI-associated diseases in the future.

## Glossary

ABCB8,: ATP-binding cassette subfamily B member 8;
ACSL4,: acyl-coA synthetase long-chain family member 4;
AIFM2,: apoptosis-inducing factor mitochondrion-associated 2;
ATF4,: activating transcription factor 4;
ARF,: alternative reading frame;
BSO,: buthionine sulfoximine
CHOP,: C/EBP homologous protein;
CoQ,: coenzyme Q;
DMT-1,: divalent metal transporter 1;
DPI2,: diphenyleneiodonium chloride 2;
ERS,: endoplasmic reticulum stress;
Fer-1,: ferrostatin-1;
FPN,: ferroportin;
FtMt,: mitochondrial ferritin;
FT,: ferritin;
FTH,: ferritin heavy chain;
FTL,: ferritin light chain;
GSH,: glutathione;
GPX4,: glutathione peroxidase 4;
HO,: hydroxyl radicals;
HO-1,: heme oxygenase;
HIF,: hypoxia-inducible factor;
HSPA5,: heat shock protein five;
IRP,: iron regulatory proteins;
IRE,: iron-responsive elements;
IRI,: ischemia-reperfusion injury;
ISCs,: iron–sulfur clusters;
IREB2,: element-binding protein 2;
LIP,: labile iron pool;
Lip-1,: liproxstatin-1;
LPCAT3,: lysophosphatidylcholine acyl transferase 3;
MIRI,: myocardial ischemia-reperfusion injury;
mTOR,: mammalian target of rapamycin;
mPTP,: mitochondrial permeability transition pore;
MCU,: mitochondrial calcium uniporter;
MFRN,: mitoferrin 2;
MMP,: mitochondrial membrane potential;
NADP-,: nicotinamide adenine dinucleotide phosphate;
NCOA4,: nuclear receptor coactivator 4; I
NOX4,: NADPH oxidase 4;
PUFAs,: polyunsaturated fatty acids;
ROS,: reactive oxygen species;
SLC7A11,: solute carrier family seven member 11;
SLC3A2,: solute carrier family three member 2;
TfR1,: transferring receptor 1;
VDAC,: voltage-dependent anion channel;

## References

[B1] Aisen P. Enns C. Wessling-Resnick M. (2001). Chemistry and Biology of Eukaryotic Iron Metabolism. Int. J. Biochem. Cel Biol. 33, 940–959. 10.1016/s1357-2725(01)00063-2 11470229

[B2] Anthonymuthu T. S. Kenny E. M. Shrivastava I. Tyurina Y. Y. Hier Z. E. Ting H.-C. (2018). Empowerment of 15-Lipoxygenase Catalytic Competence in Selective Oxidation of Membrane ETE-PE to Ferroptotic Death Signals, HpETE-PE. J. Am. Chem. Soc. 140, 17835–17839. 10.1021/jacs.8b09913 30525572 PMC6622169

[B3] Baba Y. Higa J. K. Shimada B. K. Horiuchi K. M. Suhara T. Kobayashi M. (2018). Protective Effects of the Mechanistic Target of Rapamycin against Excess Iron and Ferroptosis in Cardiomyocytes. Am. J. Physiol. Heart Circulatory Physiol. 314, H659–H668. 10.1152/ajpheart.00452.2017 PMC5899260 29127238

[B4] Bai T. Liang R. Zhu R. Wang W. Zhou L. Sun Y. (2020). MicroRNA‐214‐3p Enhances Erastin‐induced Ferroptosis by Targeting ATF4 in Hepatoma Cells. J. Cel Physiol. 235, 5637–5648. 10.1002/jcp.29496 31960438

[B5] Bayeva M. Khechaduri A. Puig S. Chang H.-C. Patial S. Blackshear P. J. (2012). mTOR Regulates Cellular Iron Homeostasis through Tristetraprolin. Cel Metab. 16, 645–657. 10.1016/j.cmet.2012.10.001 PMC3594686 23102618

[B6] Bell R. M. Yellon D. M. (2011). There Is More to Life Than Revascularization: Therapeutic Targeting of Myocardial Ischemia/reperfusion Injury. Cardiovasc. Ther. 29, e67–e79. 10.1111/j.1755-5922.2010.00190.x 20645988

[B7] Berliner J. (2002). Lipid Oxidation Products and Atherosclerosis. Vasc. Pharmacol. 38, 187–191. 10.1016/S1537-1891(02)00168-4 12449014

[B8] Boag S. E. Andreano E. Spyridopoulos I. (2017). Lymphocyte Communication in Myocardial Ischemia/reperfusion Injury. Antioxid. Redox Signal. 26, 660–675. 10.1089/ars.2016.6940 28006953

[B9] Boonnoy P. Karttunen M. Wong-Ekkabut J. (2017). Alpha-tocopherol Inhibits Pore Formation in Oxidized Bilayers. Phys. Chem. Chem. Phys. 19, 5699–5704. 10.1039/c6cp08051k 28138670

[B10] Cadenas S. (2018). ROS and Redox Signaling in Myocardial Ischemia-Reperfusion Injury and Cardioprotection. Free Radic. Biol. Med. 117, 76–89. 10.1016/j.freeradbiomed.2018.01.024 29373843

[B11] Campanella A. Rovelli E. Santambrogio P. Cozzi A. Taroni F. Levi S. (2009). Mitochondrial Ferritin Limits Oxidative Damage Regulating Mitochondrial Iron Availability: Hypothesis for a Protective Role in Friedreich Ataxia. Hum. Mol. Genet. 18, 1–11. 10.1093/hmg/ddn308 18815198 PMC3298861

[B12] Cao J. Y. Dixon S. J. (2016). Mechanisms of Ferroptosis. Cell. Mol. Life Sci. 73, 2195–2209. 10.1007/s00018-016-2194-1 27048822 PMC4887533

[B13] Chan S. Lian Q. Chen M.-P. Jiang D. Ho J. T. K. Cheung Y.-F. (2018). Deferiprone Inhibits Iron Overload-Induced Tissue Factor Bearing Endothelial Microparticle Generation by Inhibition Oxidative Stress Induced Mitochondrial Injury, and Apoptosis. Toxicol. Appl. Pharmacol. 338, 148–158. 10.1016/j.taap.2017.11.005 29132816

[B14] Chang H. C. Wu R. Shang M. Sato T. Chen C. Shapiro J. S. (2016). Reduction in Mitochondrial Iron Alleviates Cardiac Damage during Injury. EMBO Mol. Med. 8, 247–267. 10.15252/emmm.201505748 26896449 PMC4772952

[B15] Chen M.-S. Wang S.-F. Hsu C.-Y. Yin P.-H. Yeh T.-S. Lee H.-C. (2017). CHAC1 Degradation of Glutathione Enhances Cystine-Starvation-Induced Necroptosis and Ferroptosis in Human Triple Negative Breast Cancer Cells via the GCN2-eIF2α-ATF4 Pathway. Oncotarget 8, 114588–114602. 10.18632/oncotarget.23055 29383104 PMC5777716

[B16] Chen D. Tavana O. Chu B. Erber L. Chen Y. Baer R. (2017). NRF2 Is a Major Target of ARF in P53-independent Tumor Suppression. Mol. Cel 68, 224–232. 10.1016/j.molcel.2017.09.009 PMC5683418 28985506

[B17] Chen X. Xu S. Zhao C. Liu B. (2019). Role of TLR4/NADPH Oxidase 4 Pathway in Promoting Cell Death through Autophagy and Ferroptosis during Heart Failure. Biochem. Biophys. Res. Commun. 516, 37–43. 10.1016/j.bbrc.2019.06.015 31196626

[B18] Conrad M. Kagan V. E. Bayir H. Pagnussat G. C. Head B. Traber M. G. (2018). Regulation of Lipid Peroxidation and Ferroptosis in Diverse Species. Genes Dev. 32, 602–619. 10.1101/gad.314674.118 29802123 PMC6004068

[B19] Díez-López C. Comín-Colet J. González-Costello J. (2018). Iron Overload Cardiomyopathy. Curr. Opin. Cardiol. 33, 334–340. 10.1097/HCO.0000000000000511 29543671

[B20] Dixon S. J. Stockwell B. R. (2014). The Role of Iron and Reactive Oxygen Species in Cell Death. Nat. Chem. Biol. 10, 9–17. 10.1038/nchembio.1416 24346035

[B21] Dixon S. J. Lemberg K. M. Lamprecht M. R. Skouta R. Zaitsev E. M. Gleason C. E. (2012). Ferroptosis: an Iron-dependent Form of Nonapoptotic Cell Death. Cell 149, 1060–1072. 10.1016/j.cell.2012.03.042 22632970 PMC3367386

[B22] Doll S. Conrad M. (2017). Iron and Ferroptosis: A Still Ill‐defined Liaison. IUBMB Life 69, 423–434. 10.1002/iub.1616 28276141

[B23] Dongó E. Hornyák I. Benkő Z. Kiss L. (2011). The Cardioprotective Potential of Hydrogen Sulfide in Myocardial Ischemia/reperfusion Injury (Review). Acta Physiol. Hungarica 98, 369–381. 10.1556/APhysiol.98.2011.4.1 22173019

[B24] Drossos G. Lazou A. Panagopoulos P. Westaby S. (1995). Deferoxamine Cardioplegia Reduces Superoxide Radical Production in Human Myocardium. Ann. Thorac. Surg. 59, 169–172. 10.1016/0003-4975(94)00726-N 7818317

[B25] Fan Z. Wirth A.-K. Chen D. Wruck C. J. Rauh M. Buchfelder M. (2017). Nrf2-Keap1 Pathway Promotes Cell Proliferation and Diminishes Ferroptosis. Oncogenesis 6, e371. 10.1038/oncsis.2017.65 28805788 PMC5608917

[B26] Fang X. Wang H. Han D. Xie E. Yang X. Wei J. (2019). Ferroptosis as a Target for protection against Cardiomyopathy. Proc. Natl. Acad. Sci. USA 116, 2672–2680. 10.1073/pnas.1821022116 30692261 PMC6377499

[B27] Friedmann Angeli J. P. Schneider M. Proneth B. Tyurina Y. Y. Tyurin V. A. Hammond V. J. (2014). Inactivation of the Ferroptosis Regulator Gpx4 Triggers Acute Renal Failure in Mice. Nat. Cel Biol. 16, 1180–1191. 10.1038/ncb3064 PMC4894846 25402683

[B28] Fuchs Y. Steller H. (2011). Programmed Cell Death in Animal Development and Disease. Cell 147, 742–758. 10.1016/j.cell.2011.10.033 22078876 PMC4511103

[B29] Fujii J. Homma T. Kobayashi S. (2019). Ferroptosis Caused by Cysteine Insufficiency and Oxidative Insult. Free Radic. Res. 54, 969–980. 10.1080/10715762.2019.1666983 31505959

[B30] Gai C. Yu M. Li Z. Wang Y. Ding D. Zheng J. (2020). Acetaminophen Sensitizing Erastin‐induced Ferroptosis via Modulation of Nrf2/heme Oxygenase‐1 Signaling Pathway in Non‐small‐cell Lung Cancer. J. Cel Physiol. 235, 3329–3339. 10.1002/jcp.29221 PMC13482668 31541463

[B31] Gammella E. Recalcati S. Cairo G. (2016). Dual Role of ROS as Signal and Stress Agents: Iron Tips the Balance in Favor of Toxic Effects. Oxid. Med. Cell Longev. 2016, 1–9. 10.1155/2016/8629024 PMC4783558 27006749

[B32] Ganguly R. Hasanally D. Stamenkovic A. Maddaford T. G. Chaudhary R. Pierce G. N. (2018). Alpha Linolenic Acid Decreases Apoptosis and Oxidized Phospholipids in Cardiomyocytes during Ischemia/reperfusion. Mol. Cel Biochem. 437, 163–175. 10.1007/s11010-017-3104-z 28634855

[B33] Gao M. Monian P. Quadri N. Ramasamy R. Jiang X. (2015). Glutaminolysis and Transferrin Regulate Ferroptosis. Mol. Cel 59, 298–308. 10.1016/j.molcel.2015.06.011 PMC4506736 26166707

[B34] Gladyshev V. N. Stadtman T. C. Hatfield D. L. Jeang K.-T. (1999). Levels of Major Selenoproteins in T Cells Decrease during HIV Infection and Low Molecular Mass Selenium Compounds Increase. Proc. Natl. Acad. Sci. 96, 835–839. 10.1073/pnas.96.3.835 9927654 PMC15311

[B35] Gordan R. Wongjaikam S. Gwathmey J. K. Chattipakorn N. Chattipakorn S. C. Xie L.-H. (2018). Involvement of Cytosolic and Mitochondrial Iron in Iron Overload Cardiomyopathy: an Update. Heart Fail. Rev. 23, 801–816. 10.1007/s10741-018-9700-5 29675595 PMC6093778

[B36] Grueter C. E. Colbran R. J. Anderson M. E. (2006). CaMKII, an Emerging Molecular Driver for Calcium Homeostasis, Arrhythmias, and Cardiac Dysfunction. J. Mol. Med. 85, 5–14. 10.1007/s00109-006-0125-6 17119905

[B37] Guan P. Wang N. (2014). Mammalian Target of Rapamycin Coordinates Iron Metabolism with Iron-Sulfur Cluster Assembly Enzyme and Tristetraprolin. Nutrition 30, 968–974. 10.1016/j.nut.2013.12.016 24976419

[B38] Haddad S. Wang Y. Galy B. Korf-Klingebiel M. Hirsch V. Baru A. M. (2017). Iron-regulatory Proteins Secure Iron Availability in Cardiomyocytes to Prevent Heart Failure. Eur. Heart J. 38, ehw333–372. 10.1093/eurheartj/ehw333 27545647

[B39] Harris I. S. Treloar A. E. Inoue S. Sasaki M. Gorrini C. Lee K. C. (2015). Glutathione and Thioredoxin Antioxidant Pathways Synergize to Drive Cancer Initiation and Progression. Cancer Cell 27, 211–222. 10.1016/j.ccell.2014.11.019 25620030

[B40] Hayano M. Yang W. S. Corn C. K. Pagano N. C. Stockwell B. R. (2016). Loss of Cysteinyl-tRNA Synthetase (CARS) Induces the Transsulfuration Pathway and Inhibits Ferroptosis Induced by Cystine Deprivation. Cell Death Differ. 23, 270–278. 10.1038/cdd.2015.93 26184909 PMC4716307

[B41] He H. Qiao Y. Zhou Q. Wang Z. Chen X. Liu D. (2019). Iron Overload Damages the Endothelial Mitochondria via the ROS/ADMA/DDAHII/eNOS/NO Pathway. Oxid. Med. Cell Longev. 2019, 1–19. 10.1155/2019/2340392 PMC6875360 31781327

[B42] Hentze M. W. Muckenthaler M. U. Galy B. Camaschella C. (2010). Two to Tango: Regulation of Mammalian Iron Metabolism. Cell 142, 24–38. 10.1016/j.cell.2010.06.028 20603012

[B43] Hirst J. (2013). Mitochondrial Complex I. Annu. Rev. Biochem. 82, 551–575. 10.1146/annurev-biochem-070511-103700 23527692

[B44] Hoes M. F. Grote Beverborg N. Kijlstra J. D. Kuipers J. Swinkels D. W. Giepmans B. N. G. (2018). Iron Deficiency Impairs Contractility of Human Cardiomyocytes through Decreased Mitochondrial Function. Eur. J. Heart Fail. 20, 910–919. 10.1002/ejhf.1154 29484788 PMC5993224

[B45] Huang F. YangXiao R. Z. Z. Xiao Z. Xie Y. Lin X. Zhu P. (2021). Targeting Ferroptosis to Treat Cardiovascular Diseases: a New Continent to Be Explored. Front. Cel Dev. Biol. 9, 737971. 10.3389/fcell.2021.737971 PMC8435746 34527678

[B46] Ichikawa Y. Bayeva M. Ghanefar M. Potini V. Sun L. Mutharasan R. K. (2012). Disruption of ATP-Binding Cassette B8 in Mice Leads to Cardiomyopathy through a Decrease in Mitochondrial Iron export. Proc. Natl. Acad. Sci. 109, 4152–4157. 10.1073/pnas.1119338109 22375032 PMC3306722

[B47] Ingold I. Berndt C. Schmitt S. Doll S. Poschmann G. Buday K. (2018). Selenium Utilization by GPX4 Is Required to Prevent Hydroperoxide-Induced Ferroptosis. Cell 172, 409–422. 10.1016/j.cell.2017.11.048 29290465

[B48] Kagan V. E. Mao G. Qu F. Angeli J. P. F. Doll S. Croix C. S. (2017). Oxidized Arachidonic and Adrenic PEs Navigate Cells to Ferroptosis. Nat. Chem. Biol. 13, 81–90. 10.1038/nchembio.2238 27842066 PMC5506843

[B49] Kakhlon O. Cabantchik Z. I. (2002). The Labile Iron Pool: Characterization, Measurement, and Participation in Cellular Processes1 1This Article Is Part of a Series of Reviews on "Iron and Cellular Redox Status." the Full List of Papers May Be Found on the Homepage of the Journal. Free Radic. Biol. Med. 33, 1037–1046. 10.1016/S0891-5849(02)01006-7 12374615

[B50] Kawabata H. (2019). Transferrin and Transferrin Receptors Update. Free Radic. Biol. Med. 133, 46–54. 10.1016/j.freeradbiomed.2018.06.037 29969719

[B51] Kerins M. J. Ooi A. (2018). The Roles of NRF2 in Modulating Cellular Iron Homeostasis. Antioxid. Redox Signal. 29, 1756–1773. 10.1089/ars.2017.7176 28793787 PMC6208163

[B52] Krainz T. Gaschler M. M. Lim C. Sacher J. R. Stockwell B. R. Wipf P. (2016). A Mitochondrial-Targeted Nitroxide Is a Potent Inhibitor of Ferroptosis. ACS Cent. Sci. 2, 653–659. 10.1021/acscentsci.6b00199 27725964 PMC5043442

[B53] Kruszewski M. (2003). Labile Iron Pool: the Main Determinant of Cellular Response to Oxidative Stress. Mutat. Res. Fund. Mol. Mech. Mutagen. 531, 81–92. 10.1016/j.mrfmmm.2003.08.004 14637247

[B54] Kuo K.-L. Hung S.-C. Lee T.-S. Tarng D.-C. (2014). Iron Sucrose Accelerates Early Atherogenesis by Increasing Superoxide Production and Upregulating Adhesion Molecules in CKD. J. Am. Soc. Nephrol. 25, 2596–2606. 10.1681/ASN.2013080838 24722448 PMC4214520

[B55] Kuroda J. Ago T. Matsushima S. Zhai P. Schneider M. D. Sadoshima J. (2010). NADPH Oxidase 4 (Nox4) Is a Major Source of Oxidative Stress in the Failing Heart. Proc. Natl. Acad. Sci. 107, 15565–15570. 10.1073/pnas.1002178107 20713697 PMC2932625

[B56] Lakhal-Littleton S. Wolna M. Chung Y. J. Christian H. C. Heather L. C. Brescia M. (2016). An Essential Cell-Autonomous Role for Hepcidin in Cardiac Iron Homeostasis. Elife 5, e19804. 10.7554/eLife.19804 27897970 PMC5176354

[B57] Lane D. Bae D.-H. Merlot A. Sahni S. Richardson D. (2015). Duodenal Cytochrome B (DCYTB) in Iron Metabolism: an Update on Function and Regulation. Nutrients 7, 2274–2296. 10.3390/nu7042274 25835049 PMC4425144

[B58] Lesnefsky E. J. Chen Q. Tandler B. Hoppel C. L. (2017). Mitochondrial Dysfunction and Myocardial Ischemia-Reperfusion: Implications for Novel Therapies. Annu. Rev. Pharmacol. Toxicol. 57, 535–565. 10.1146/annurev-pharmtox-010715-103335 27860548 PMC11060135

[B59] Li J. Zhao Y. Zhou N. Li L. Li K. (2019). Dexmedetomidine Attenuates Myocardial Ischemia-Reperfusion Injury in Diabetes Mellitus by Inhibiting Endoplasmic Reticulum Stress. J. Diabetes Res. 2019, 1–12. 10.1155/2019/7869318 PMC6914963 31886285

[B60] Li W. Li W. Leng Y. Xiong Y. Xia Z. (2020). Ferroptosis Is Involved in Diabetes Myocardial Ischemia/reperfusion Injury through Endoplasmic Reticulum Stress. DNA Cel Biol. 39, 210–225. 10.1089/dna.2019.5097 31809190

[B61] Liu Y. Wang W. Li Y. Xiao Y. Cheng J. Jia J. (2015). The 5-lipoxygenase Inhibitor Zileuton Confers Neuroprotection against Glutamate Oxidative Damage by Inhibiting Ferroptosis. Biol. Pharm. Bull. 38, 1234–1239. 10.1248/bpb.b15-00048 26235588

[B62] Louandre C. Marcq I. Bouhlal H. Lachaier E. Godin C. Saidak Z. (2015). The Retinoblastoma (Rb) Protein Regulates Ferroptosis Induced by Sorafenib in Human Hepatocellular Carcinoma Cells. Cancer Lett. 356, 971–977. 10.1016/j.canlet.2014.11.014 25444922

[B63] Magtanong L. Ko P.-J. To M. Cao J. Y. Forcina G. C. Tarangelo A. (2019). Exogenous Monounsaturated Fatty Acids Promote a Ferroptosis-Resistant Cell State. Cel Chem. Biol. 26, 420–432. 10.1016/j.chembiol.2018.11.016 PMC6430697 30686757

[B64] Maldonado E. N. Sheldon K. L. DeHart D. N. Patnaik J. Manevich Y. Townsend D. M. (2013). Voltage-dependent Anion Channels Modulate Mitochondrial Metabolism in Cancer Cells. J. Biol. Chem. 288, 11920–11929. 10.1074/jbc.M112.433847 23471966 PMC3636879

[B65] Marshall K. R. Gong M. Wodke L. Lamb J. H. Jones D. J. L. Farmer P. B. (2005). The Human Apoptosis-Inducing Protein AMID Is an Oxidoreductase with a Modified Flavin Cofactor and DNA Binding Activity. J. Biol. Chem. 280, 30735–30740. 10.1074/jbc.M414018200 15958387

[B66] Matsushima S. Tsutsui H. Sadoshima J. (2014). Physiological and Pathological Functions of NADPH Oxidases during Myocardial Ischemia-Reperfusion. Trends Cardiovasc. Med. 24, 202–205. 10.1016/j.tcm.2014.03.003 24880746 PMC4119873

[B67] Morciano G. Giorgi C. Bonora M. Punzetti S. Pavasini R. Wieckowski M. R. (2015). Molecular Identity of the Mitochondrial Permeability Transition Pore and its Role in Ischemia-Reperfusion Injury. J. Mol. Cell Cardiol. 78, 142–153. 10.1016/j.yjmcc.2014.08.015 25172387

[B68] Mou Y. Wang J. Wu J. He D. Zhang C. Duan C. (2019). Ferroptosis, a New Form of Cell Death: Opportunities and Challenges in Cancer. J. Hematol. Oncol. 12, 34. 10.1186/s13045-019-0720-y 30925886 PMC6441206

[B69] Ng S.-W. Norwitz S. G. Taylor H. S. Norwitz E. R. (2020). Endometriosis: the Role of Iron Overload and Ferroptosis. Reprod. Sci. 27, 1383–1390. 10.1007/s43032-020-00164-z 32077077

[B70] Nie G. Sheftel A. D. Kim S. F. Ponka P. (2005). Overexpression of Mitochondrial Ferritin Causes Cytosolic Iron Depletion and Changes Cellular Iron Homeostasis. Blood 105, 2161–2167. 10.1182/blood-2004-07-2722 15522954

[B71] Omiya S. Hikoso S. Imanishi Y. Saito A. Yamaguchi O. Takeda T. (2009). Downregulation of Ferritin Heavy Chain Increases Labile Iron Pool, Oxidative Stress and Cell Death in Cardiomyocytes. J. Mol. Cell Cardiol. 46, 59–66. 10.1016/j.yjmcc.2008.09.714 18992754

[B72] Oudit G. Y. Sun H. Trivieri M. G. Koch S. E. Dawood F. Ackerley C. (2003). L-type Ca2+ Channels Provide a Major Pathway for Iron Entry into Cardiomyocytes in Iron-Overload Cardiomyopathy. Nat. Med. 9, 1187–1194. 10.1038/nm920 12937413

[B73] Paraskevaidis I. A. Iliodromitis E. K. Vlahakos D. Tsiapras D. P. Nikolaidis A. Marathias A. (2005). Deferoxamine Infusion during Coronary Artery Bypass Grafting Ameliorates Lipid Peroxidation and Protects the Myocardium against Reperfusion Injury: Immediate and Long-Term Significance. Eur. Heart J. 26, 263–270. 10.1093/eurheartj/ehi028 15618054

[B74] Paterek A. Mackiewicz U. Mączewski M. (2019). Iron and the Heart: a Paradigm Shift from Systemic to Cardiomyocyte Abnormalities. J. Cel. Physiol. 234, 21613–21629. 10.1002/jcp.28820 31106422

[B75] Paul B. T. Manz D. H. Torti F. M. Torti S. V. (2017). Mitochondria and Iron: Current Questions. Expert Rev. Hematol. 10, 65–79. 10.1080/17474086.2016.1268047 27911100 PMC5538026

[B76] Pell V. R. Chouchani E. T. Frezza C. Murphy M. P. Krieg T. (2016). Succinate Metabolism: a New Therapeutic Target for Myocardial Reperfusion Injury. Cardiovasc. Res. 111, 134–141. 10.1093/cvr/cvw100 27194563

[B77] Pitale P. M. Gorbatyuk O. Gorbatyuk M. (2017). Neurodegeneration: Keeping ATF4 on a Tight Leash. Front. Cel. Neurosci. 11, 410. 10.3389/fncel.2017.00410 PMC5736573 29326555

[B78] Richardson D. R. Lane D. J. R. Becker E. M. Huang M. L. H. Whitnall M. Rahmanto Y. S. (2010). Mitochondrial Iron Trafficking and the Integration of Iron Metabolism between the Mitochondrion and Cytosol. Proc. Natl. Acad. Sci. 107, 10775–10782. 10.1073/pnas.0912925107 20495089 PMC2890738

[B79] Russo I. Penna C. Musso T. Popara J. Alloatti G. Cavalot F. (2017). Platelets, Diabetes and Myocardial Ischemia/reperfusion Injury. Cardiovasc. Diabetol. 16, 71. 10.1186/s12933-017-0550-6 28569217 PMC5452354

[B80] Santambrogio P. Biasiotto G. Sanvito F. Olivieri S. Arosio P. Levi S. (2007). Mitochondrial Ferritin Expression in Adult Mouse Tissues. J. Histochem. Cytochem. 55, 1129–1137. 10.1369/jhc.7A7273.2007 17625226 PMC3957534

[B81] Schanze N. Bode C. Duerschmied D. (2019). Platelet Contributions to Myocardial Ischemia/reperfusion Injury. Front. Immunol. 10, 1260. 10.3389/fimmu.2019.01260 31244834 PMC6562336

[B82] Sehm T. Fan Z. Ghoochani A. Rauh M. Engelhorn T. Minakaki G. (2016). Sulfasalazine Impacts on Ferroptotic Cell Death and Alleviates the Tumor Microenvironment and Glioma-Induced Brain Edema. Oncotarget 7, 36021–36033. 10.18632/oncotarget.8651 27074570 PMC5094980

[B83] Sharma A. Flora S. J. S. (2021). Positive and Negative Regulation of Ferroptosis and its Role in Maintaining Metabolic and Redox Homeostasis. Oxid. Med. Cell Longev. 2021, 1–13. 10.1155/2021/9074206 PMC8102094 34007410

[B84] Shaw A. T. Winslow M. M. Magendantz M. Ouyang C. Dowdle J. Subramanian A. (2011). Selective Killing of K-Ras Mutant Cancer Cells by Small Molecule Inducers of Oxidative Stress. Proc. Natl. Acad. Sci. 108, 8773–8778. 10.1073/pnas.1105941108 21555567 PMC3102385

[B85] Skouta R. Dixon S. J. Wang J. Dunn D. E. Orman M. Shimada K. (2014). Ferrostatins Inhibit Oxidative Lipid Damage and Cell Death in Diverse Disease Models. J. Am. Chem. Soc. 136, 4551–4556. 10.1021/ja411006a 24592866 PMC3985476

[B86] Sripetchwandee J. Kenknight S. B. Sanit J. Chattipakorn S. Chattipakorn N. (2014). Blockade of Mitochondrial Calcium Uniporter Prevents Cardiac Mitochondrial Dysfunction Caused by Iron Overload. Acta Physiol. 210, 330–341. 10.1111/apha.12162 24034353

[B87] Stamenkovic A. O’Hara K. A. Nelson D. C. Maddaford T. G. Edel A. L. Maddaford G. (2021). Oxidized Phosphatidylcholines Trigger Ferroptosis in Cardiomyocytes during Ischemia-Reperfusion Injury. Am. J. Physiol. Heart Circulatory Physiol. 320, H1170–H1184. 10.1152/ajpheart.00237.2020 33513080

[B88] Stockwell B. R. Friedmann Angeli J. P. Bayir H. Bush A. I. Conrad M. Dixon S. J. (2017). Ferroptosis: a Regulated Cell Death Nexus Linking Metabolism, Redox Biology, and Disease. Cell 171, 273–285. 10.1016/j.cell.2017.09.021 28985560 PMC5685180

[B89] Sullivan J. L. (2009). Iron in Arterial Plaque: A Modifiable Risk Factor for Atherosclerosis. Biochim. Biophys. Acta Gen. Subjects 1790, 718–723. 10.1016/j.bbagen.2008.06.005 18619522

[B90] Sun X. Ou Z. Chen R. Niu X. Chen D. Kang R. (2016). Activation of the P62-Keap1-NRF2 Pathway Protects against Ferroptosis in Hepatocellular Carcinoma Cells. Hepatology 63, 173–184. 10.1002/hep.28251 26403645 PMC4688087

[B91] Sun X. Yang Y. Shi J. Wang C. Yu Z. Zhang H. (2017). NOX4- and Nrf2-Mediated Oxidative Stress Induced by Silver Nanoparticles in Vascular Endothelial Cells. J. Appl. Toxicol. 37, 1428–1437. 10.1002/jat.3511 28815642

[B92] Tang W. H. Wu S. Wong T. M. Chung S. K. Chung S. S. M. (2008). Polyol Pathway Mediates Iron-Induced Oxidative Injury in Ischemic-Reperfused Rat Heart. Free Radic. Biol. Med. 45, 602–610. 10.1016/j.freeradbiomed.2008.05.003 18549825

[B93] Tateda C. Kusano T. Takahashi Y. (2012). The Arabidopsis Voltage-dependent Anion Channel 2 Is Required for Plant Growth. Plant Signal. Behav. 7, 31–33. 10.4161/psb.7.1.18394 22301963 PMC3357362

[B94] Tian L. Cao W. Yue R. Yuan Y. Guo X. Qin D. (2019). Pretreatment with Tilianin Improves Mitochondrial Energy Metabolism and Oxidative Stress in Rats with Myocardial Ischemia/reperfusion Injury via AMPK/SIRT1/PGC-1 Alpha Signaling Pathway. J. Pharmacol. Sci. 139, 352–360. 10.1016/j.jphs.2019.02.008 30910451

[B95] Turer A. T. Hill J. A. (2010). Pathogenesis of Myocardial Ischemia-Reperfusion Injury and Rationale for Therapy. Am. J. Cardiol. 106, 360–368. 10.1016/j.amjcard.2010.03.032 20643246 PMC2957093

[B96] Valko M. Jomova K. Rhodes C. J. Kuča K. Musílek K. (2016). Redox- and Non-redox-metal-induced Formation of Free Radicals and Their Role in Human Disease. Arch. Toxicol. 90, 1–37. 10.1007/s00204-015-1579-5 26343967

[B97] Vela D. (2020). Keeping Heart Homeostasis in Check through the Balance of Iron Metabolism. Acta Physiol. 228, e13324. 10.1111/apha.13324 31162883

[B98] Verkhratsky A. Parpura V. (2014). Calcium Signalling and Calcium Channels: Evolution and General Principles. Eur. J. Pharmacol. 739, 1–3. 10.1016/j.ejphar.2013.11.013 24291103 PMC4037395

[B99] Vinten-Johansen J. Jiang R. Reeves J. G. Mykytenko J. Deneve J. Jobe L. J. (2007). Inflammation, Proinflammatory Mediators and Myocardial Ischemia-Reperfusion Injury. Hematol. Oncol. Clin. North Am. 21, 123–145. 10.1016/j.hoc.2006.11.010 17258123

[B100] Wang Y.-Q. Chang S.-Y. Wu Q. Gou Y.-J. Jia L. Cui Y.-M. (2016). The Protective Role of Mitochondrial Ferritin on Erastin-Induced Ferroptosis. Front. Aging Neurosci. 8, 308. 10.3389/fnagi.2016.00308 28066232 PMC5167726

[B101] Wang N. Zeng G.-Z. Yin J.-L. Bian Z.-X. (2019). Artesunate Activates the ATF4-CHOP-CHAC1 Pathway and Affects Ferroptosis in Burkitt's Lymphoma. Biochem. Biophys. Res. Commun. 519, 533–539. 10.1016/j.bbrc.2019.09.023 31537387

[B102] Watt R. K. (2013). A Unified Model for Ferritin Iron Loading by the Catalytic center: Implications for Controlling “Free Iron” during Oxidative Stress. ChemBioChem 14, 415–419. 10.1002/cbic.201200783 23404831

[B103] White C. W. Hasanally D. Mundt P. Li Y. Xiang B. Klein J. (2015). A Whole Blood-Based Perfusate Provides superior Preservation of Myocardial Function during *Ex Vivo* Heart Perfusion. J. Heart Lung Transplant. 34, 113–121. 10.1016/j.healun.2014.09.021 25447577

[B104] Williams R. E. Zweier J. L. Flaherty J. T. (1991). Treatment with Deferoxamine during Ischemia Improves Functional and Metabolic Recovery and Reduces Reperfusion-Induced Oxygen Radical Generation in Rabbit Hearts. Circulation 83, 1006–1014. 10.1161/01.cir.83.3.1006 1847847

[B105] Wofford J. D. Chakrabarti M. Lindahl P. A. (2017). Mössbauer Spectra of Mouse Hearts Reveal Age-dependent Changes in Mitochondrial and Ferritin Iron Levels. J. Biol. Chem. 292, 5546–5554. 10.1074/jbc.M117.777201 28202542 PMC5392696

[B106] Wood J. C. (2008). Cardiac Iron across Different Transfusion-dependent Diseases. Blood Rev. 22 (Suppl. 2), S14–S21. 10.1016/S0268-960X(08)70004-3 19059052 PMC2896332

[B107] Wu W. Chang S. Wu Q. Xu Z. Wang P. Li Y. (2016). Mitochondrial Ferritin Protects the Murine Myocardium from Acute Exhaustive Exercise Injury. Cell Death Dis 7, e2475. 10.1038/cddis.2016.372 27853170 PMC5260894

[B108] Xie Y. Hou W. Song X. Yu Y. Huang J. Sun X. (2016). Ferroptosis: Process and Function. Cel Death Differ. 23, 369–379. 10.1038/cdd.2015.158 PMC5072448 26794443

[B109] Yagoda N. von Rechenberg M. Zaganjor E. Bauer A. J. Yang W. S. Fridman D. J. (2007). RAS-RAF-MEK-dependent Oxidative Cell Death Involving Voltage-dependent Anion Channels. Nature 447, 865–869. 10.1038/nature05859 PMC3047570 17568748

[B110] Yang W. S. SriRamaratnam R. Welsch M. E. Shimada K. Skouta R. Viswanathan V. S. (2014). Regulation of Ferroptotic Cancer Cell Death by GPX4. Cell 156, 317–331. 10.1016/j.cell.2013.12.010 24439385 PMC4076414

[B111] Yang F. Qin Y. Lv J. Wang Y. Che H. Chen X. (2018). Silencing Long Non-coding RNA Kcnq1ot1 Alleviates Pyroptosis and Fibrosis in Diabetic Cardiomyopathy. Cel Death Dis. 9, 1000. 10.1038/s41419-018-1029-4 PMC6155223 30250027

[B112] Yeang C. Hasanally D. Que X. Hung M.-Y. Stamenkovic A. Chan D. (2019). Reduction of Myocardial Ischaemia-Reperfusion Injury by Inactivating Oxidized Phospholipids. Cardiovasc. Res. 115, 179–189. 10.1093/cvr/cvy136 29850765 PMC6302283

[B113] Yin H. Xu L. Porter N. A. (2011). Free Radical Lipid Peroxidation: Mechanisms and Analysis. Chem. Rev. 111, 5944–5972. 10.1021/cr200084z 21861450

[B114] Zhang L. Gong F. Zhang F. Ma J. Zhang P. D. Shen J. (2013). Targeted Therapy for Human Hepatic Carcinoma Cells Using Folate-Functionalized Polymeric Micelles Loaded with Superparamagnetic Iron Oxide and Sorafenib *In Vitro* . Int. J. Nanomedicine 8, 1517–1524. 10.2147/IJN.S43263 23620667 PMC3633582

[B115] Zhang Y. Liu D. Hu H. Zhang P. Xie R. Cui W. (2019). HIF-1α/BNIP3 Signaling Pathway-Induced-Autophagy Plays Protective Role during Myocardial Ischemia-Reperfusion Injury. Biomed. Pharmacother. 120, 109464. 10.1016/j.biopha.2019.109464 31590128

[B116] Zhang Y. Sun C. Zhao C. Hao J. Zhang Y. Fan B. (2019). Ferroptosis Inhibitor SRS 16-86 Attenuates Ferroptosis and Promotes Functional Recovery in Contusion Spinal Cord Injury. Brain Res. 1706, 48–57. 10.1016/j.brainres.2018.10.023 30352209

[B117] Zhou T. Chuang C.-C. Zuo L. (2015). Molecular Characterization of Reactive Oxygen Species in Myocardial Ischemia-Reperfusion Injury. Biomed. Res. Int. 2015, 1–9. 10.1155/2015/864946 PMC4609796 26509170

[B118] Zhu S. Zhang Q. Sun X. Zeh H. J. Lotze M. T. Kang R. (2017). HSPA5 Regulates Ferroptotic Cell Death in Cancer Cells. Cancer Res. 77, 2064–2077. 10.1158/0008-5472.CAN-16-1979 28130223 PMC5392369

[B119] Zilka O. Shah R. Li B. Friedmann Angeli J. P. Griesser M. Conrad M. (2017). On the Mechanism of Cytoprotection by Ferrostatin-1 and Liproxstatin-1 and the Role of Lipid Peroxidation in Ferroptotic Cell Death. ACS Cent. Sci. 3, 232–243. 10.1021/acscentsci.7b00028 28386601 PMC5364454

